# Immunoregulatory Role of the Mechanosensitive Ion Channel Piezo1 in Inflammation and Cancer

**DOI:** 10.3390/molecules28010213

**Published:** 2022-12-26

**Authors:** Yuexin Wang, Zhiyuan Zhang, Qiuli Yang, Yejin Cao, Yingjie Dong, Yujing Bi, Guangwei Liu

**Affiliations:** 1 Key Laboratory of Cell Proliferation and Regulation Biology, Ministry of Education, College of Life Sciences, Beijing Normal University, Beijing 100875, China; 2 State Key Laboratory of Pathogen and Biosecurity, Beijing Institute of Microbiology and Epidemiology, Beijing 100071, China

**Keywords:** Piezo1, mechanosensitive ion channel, immunity, inflammation, cancer, cancer immunotherapy, virus infection, lung

## Abstract

Piezo1 was originally identified as a mechanically activated, nonselective cation ion channel, with significant permeability to calcium ions, is evolutionally conserved, and is involved in the proliferation and development of various types of cells, in the context of various types of mechanical or innate stimuli. Recently, our study and work by others have reported that Piezo1 from all kinds of immune cells is involved in regulating many diseases, including infectious inflammation and cancer. This review summarizes the recent progress made in understanding the immunoregulatory role and mechanisms of the mechanical receptor Piezo1 in inflammation and cancer and provides new insight into the biological significance of Piezo1 in regulating immunity and tumors.

## 1. Introduction

In nature, organisms are constantly stimulated by mechanical stimuli from the outside environment, during their development and growth. It is essential for the organism to feel external mechanical forces and to respond to them, to maintain the normal physiological function of the internal environment. Mechanical conductance is a fundamental biological process for the transmission of signals, which encourages eukaryotic or prokaryotic cells to convert mechanical force signals to electrochemical signals. Ion channels are the major receptors that mediate the rapid response of mechanical force signals. Ion channels regulate the concentration of ions inside and outside the cell membrane, by facilitating the transmembrane transport of various inorganic ions. The ion channels are divided into mechanically gated ion channels, ligand-gated ion channels, and voltage-gated ion channels, of which some examples are exhibited in Table 1 [1]. Mechanosensitive ion channels can detect changes of mechanical force on the cell membrane and respond in a few microseconds. When the channels are opened with a mechanical sensor, the signals of mechanical stimulation will be transformed into electrical and chemical signals in the cells [2]. Mechanosensitive channels of ions are widely distributed in various tissues and organs of the body, and regulate basic physiological processes, such as touch, pain, and blood pressure [3]. However, in the past few decades, scientists have not yet understood the molecular mechanism of mechanosensitive ion channels in organisms. In 2010, Professor Ardem Patapoutian’s group at the Scripps Research Institute in California discovered a kind of pressure protein named Piezo that can mediate mechanical transduction in mammals for the first time [4]. The family of Piezo channels in mammals is divided into two subtypes, including the Piezo1 and Piezo2 molecules, which are the same types and structures responsible for regulating the flow of movement of ions from mechanically active cells into or out of the cells. Between them, Piezo1, made up of 2500 amino acids, with a trimeric propeller-like structure, was first identified in the mouse brain. Piezo1 is related to pathophysiological processes, such as the development of the vascular system, the remodeling of the arteries, and the regulation of blood pressure [5]. Piezo2 is mainly involved in proprioception and in the induction of light sensitivity [6]. In this review, we investigate the research progress of Piezo1 in the immunomodulatory mechanism, and Piezo1 has profound effects on inflammation and cancer.

## 2. The Biological Effect of Piezo1

**Figure 1:**
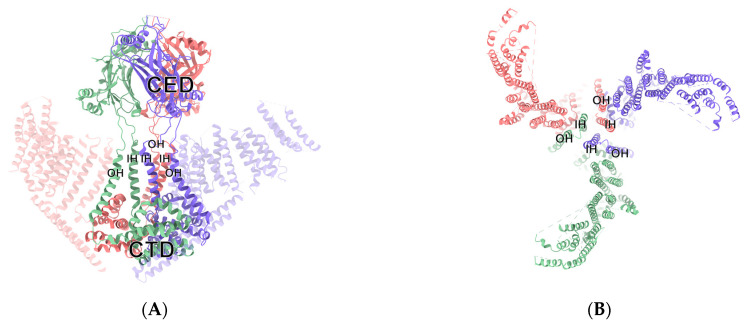
Structure of Piezo 1 channel protein (PDB: 3JAC). (**A**) The main view of Piezo1 protein. For a clear view of the channel, some domains outside the channel core are transparentized or hidden. (**B**) A view from the top of Piezo1 protein (central sliced). Domains which construct the channel core are labeled in A and B.

Under the electron microscope, Piezo1 channel protein exhibits a trimer-like three-bladed propeller structure composed of a central part and peripheral part, the dominant ion conduction hole module, and three highly curved nonplanar blades [7]. The central channel is composed of approximately 350 amino acids at the end of a hydroxyl group, including the inner helix (IH), C-terminal extracellular domain (CED), outer helix (OH), and intracellular C-terminal domain (CTD) [8]. The characteristic peripheral domain is composed of 2200 amino acids in its amino terminus, including three regions: “anchor”, “beam”, and “blade”. The long rod is connected to the region of the blade with the intracellular C-terminal domain and the region of the anchor domain of central channel regions, thereby allowing the mechanical forces of the blade to be effectively transmitted to the central channel part, by the principle of the lever [9]. When stimulated by external mechanical forces, a number of reversible changes occur in the conformation of the Piezo1 channel protein [10]. By means of a lever-like device, formed by changing the structure, Piezo1 delivers the conformation changes formed on the distal blade, which results in a change in the open surface of the pore of the ions, which allows the passage of cations and depolarizes the cell membrane, as shown in Figure 1 [8]. In addition, Piezo1 can be opened in the absence of mechanical stimulation. New research suggests that there is a specific allosteric site for the chemical binding of the Piezo1 channel that could bind with the specific agonist of a Piezo1 small-molecule agonist (Yoda1), and activate Piezo1 in the absence of mechanical stimulation. Recent advances in cryo-electron microscopy, for example, have made it possible to trap mouse Piezo1 in a non-conducting state, that may correspond to a regular closed conformation, formed in the absence of an external force. This provides the theoretical basis for the identification of a target drug for the clinical treatment of Piezo1-related diseases [11].

Recent studies have shown that Piezio1, which is expressed in a number of mechanosensory organs, such as the lungs, blood vessels, and bones, controls cell reproduction and adhesion, and regulates the differentiation and migration of cells [12,13]. The channel protein Piezo1 plays an essential role in the development of blood vessels, vascular remodeling, and the regulation of blood pressure [14]. The Piezo1 channel is primarily responsible for the integration of blood vessels in the body. The absence of Piezo1 throughout the body, particularly in the endothelial cells of mice, severely interferes with the proliferation and development of blood vessels. Knock-out of Piezo1 in the second trimester of developing mice can trigger the death of the embryos. Haploinsufficiency does not kill, but abnormal vascular endothelial cells are still found in the blood vessels of mature mice. The Piezo1 channel protein regulates the spatial organization of endothelial cells by controlling calcium influx into the cell, which in turn activates the downstream activity of proteases [15]. In the case of vasodilation, Piezo1 is strongly expressed in the muscle cells and in arterioles, and is implicated in the remodeling of the arteries. Piezo1 enhances the expression of calcium and stimulates the upregulation of transglutaminase, which in hypertensive patients alters the diameter and changes the thickness of the wall of the resistance artery [16]. In the regulation of blood pressure, Piezo1, the mechanically sensitive cationic channel in the endothelium, facilitates a mechanically activated release of the adrenal medullary and activates its Gs-coupled receptor, thus deregulating the expression of cytoplasmic cyclic adenosine monophosphate (cAMP). This promotes protein kinase A (PKA)-induced endothelial NO synthase (eNOS) phosphorylation at Serine 633, leading to the activation of eNOS and vasodilation [17]. The fluid shearing stress exerted by the flow of blood is applicable to the vascular endothelium, which turns the mechanical stimulation signals of laminar flow into atherosclerotic protective signals and inflammatory signals, induced by disturbances in the atherosclerotic region. The endothelium-specific Piezo1 knockout in mice showed the effects of inflammation of the endothelia and decreased progression of atherosclerosis in the atherosclerotic region. This offers a new strategy for treating inflammatory vascular diseases [18].

Under different types and conditions of mechanical stimulation and different effects on the environment, activated Piezo1 has different effects. The lungs are stimulated constantly by hemodynamic and respiratory mechanics, by the sensation of pressure in the veins, the pressure in the blood flow, and the contraction of circulation. Piezo1 is abundant in lung cells and is a part of the physiological and pathological functions of the lung [4]. Pulmonary edema is caused by the breakdown of the pulmonary endothelial barrier and the migration and recruitment of plasma proteins in the pulmonary capillaries. Although there are a few adhesion molecules and receptors, most of the mechanisms currently known involve vascular endothelial adherence, which is an important adhesion molecule that maintains the stability of endothelial connections, and is involved in the regulation of endothelial cell contact, the control of vascular wall barrier function, and the control of leukocyte exostosis. The inhibition of VE-cadherin may disrupt the equilibrium of the environmental homeostasis of pulmonary fluid [19]. Piezo1 plays a major role in sensing increased pressure on lung microstructures and the destruction of endothelial barriers. Conditional demolition of Piezo1 by endothelial cells (ECs) in mice was shown to trigger pressure on the lung vasculature and break down the opening of the lung endothelial barrier. Piezo1 signals abnormal permeability of lung blood vessels by contributing to the degradation of the endothelial adhesion junction (AJ) protein, VE-cadherin. A suppressed expression of Piezo1 could limit the damage caused by a stress-induced collapse of the lung capillaries in response to a rise in blood pressure [20]. Mechanical stimulation of lung endothelial cells by Piezo1 leads to Ca^2+^ influx, which in turn activates downstream target calpain, to increase the amount of lipopolysaccharide (LPS), thereby promoting further damage to the lung vascular barrier and hyperpermeability of the vasculature. Piezo1, a key mediator of the lung endothelial barrier, may play a role in regulating lung damage caused by acute respiratory distress syndrome (ARDS), which is caused by a large expansion of the alveolus and a heightened tension of the endothelial cell membrane, in the continual circulation of living organisms [21]. As a load-bearing organ, bone is a biological scaffold which is under constant pressure from both the internal and external environment. Mechanical loading of skeletal systems is important for the development and maintenance of bone. Piezo1 plays a key role in the formation of bone and in the knockout of osteoblastic cells. Piezo1 severely damages the structure and strength of bones. The generally low expression of Piezo1 in patients with osteoporosis suggests that Piezo1 could be a new drug target for this disease, a key sensor for the mechanical sensitivities of osteoblasts [22].

In conclusion, the Piezo1 protein is involved in the regulation of proliferation, differentiation, adhesion, migration, and apoptosis in different tissues and in different organs of the body, by detecting mechanical stimulation signals and organs throughout the body, by sensing mechanical stimulation signals, playing a crucial role in the normal life of the organism and in the surveillance of disease.

## 3. The Role of Piezo1 in Innate Immunity

**Figure 2:**
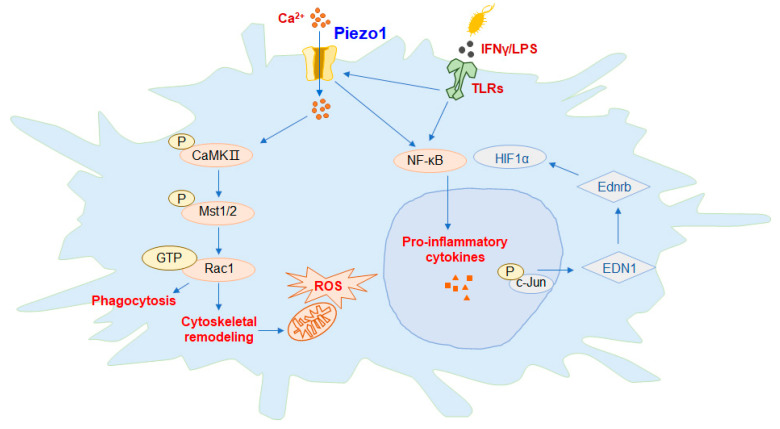
Piezo1 regulates the function of macrophages. In macrophages, activation of Piezo1 channel proteins can promote the expression of downstream proteins and the phagocytic activity of macrophages, polarizing the cells to the M1 phenotype. Figure 2 summarizes the available mechanisms by which Piezo1 activates macrophages, including activation of Piezo1, either directly or through interactions with toll-like receptor (TLR), controls Ca^2+^ transport into the cell, promotes endothelin-1 (EDN1) expression, induces nuclear translocation of hypoxia irreducible Factor 1α (HIF1α) and nuclear factor kappa-B (NF-κB), and enables massive extracellular secretion of the proinflammatory cytokines interleukin 6 (IL-6) and tumor necrosis factor-α (TNF-α). The feedback loop between Piezo1 and actin polymerization also has important implications for macrophage function. The phosphorylation of key mammalian STE20-like protein kinases 1 (MST1) and MST2 in the calmodulin (CaM)-dependent kinases II (CaMKII) and Hippo signaling pathways is activated, then acts on the Rac signaling pathway to regulate the release of reactive oxygen species (ROS) by mitochondria and promote the phagocytosis and antibacterial ability of macrophages.

**Figure 3:**
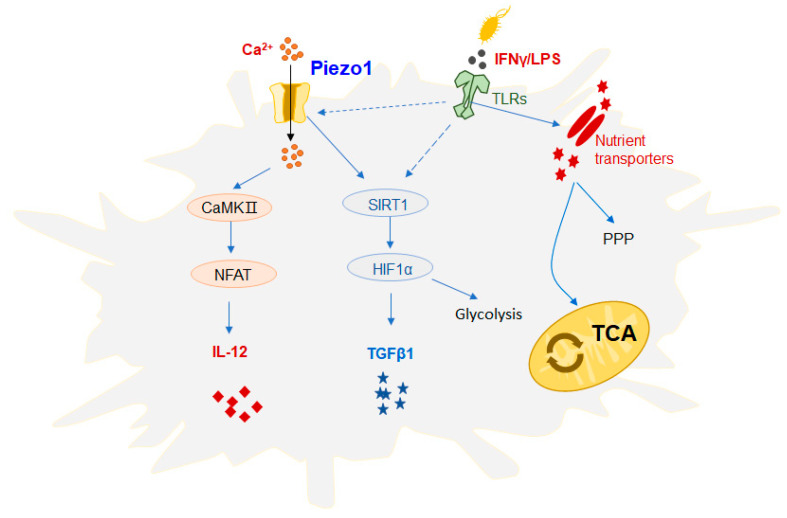
Effect of Piezo1 on the function of DCs. Mechanical stimulation can activate Piezo1 signaling, regulate dendritic cell (DC) responses, and trigger inflammatory responses. Recent studies have demonstrated that DC cells growing at a high rigidity in vitro show significantly increased proliferation, activation cytokine production, and a significant upregulation of glucose metabolism pathway flux, including the tricarboxylic acid cycle (TCA cycle) and pentose phosphate pathway (PPP). In addition, studies have found that Piezo1 can integrate the SIRT1-HIF1α glucose metabolism pathway and the Ca^2+^-calmodulin-nuclear transcription factor (NFAT) pathway to govern the secretion of DC cytokines. The DC mechanization of Piezo1 stimulated by mechanical force or inflammatory signaling regulates the mutual differentiation of Th1 and regulatory T cells (Treg cells) in the inhibition of tumor growth. Piezo1 is present in response to both inflammation and mechanical stimulation, which can mediate changes in ion concentration and metabolic signals in the cellular environment and alter its function, thus playing an important role in disease progression.

Current studies have demonstrated that the ability to perceive and respond to immune cells, in response to the mechanical stimulus of the external environment, is a significant part of the daily physiological responses of the organism. Both macrophages and dendritic cells (DCs) in innate immunity cells can respond to a few common mechanisms to mechanical stimuli (Figure 2 and Figure 3). The physiological role of Piezo1, the mechanically sensitive ion channel, is discussed above. Considering the mechanism by which the innate immune system responds to mechanically sensitive signals can provide potential drug targets for the subsequent treatment of a variety of diseases, such as autoimmune diseases, infections, and malignant tumors.

In macrophages, activation of the Piezo1 channel protein by mechanical stimulation of the gene alters the expression and activity of phagocytes, and macrophages are predisposed to the formation of classically activated macrophages (M1), which are a type of proinflammatory macrophage (Figure 2). Recent research suggests that the cationic channel protein Piezo1, activated by reflex stimulation signals, plays an important role in sensing the polarization of macrophages and the stiffness of microenvironments. Mice macrophages with Piezo1 knocked out show an increase in the suppression of inflammation and the healing of wounds. Interferon-γ (IFN-γ)/LPS activates the Piezo1-mediated increase in Ca^2+^ influx in severe exacerbation, thereby promoting the polymerization of actin. Piezo1 enhances inflammation by regulating NF-κB and proinflammatory markers, including tumor necrosis factor-α (TNF-α) and interleukin 6 (IL-6). Piezo1 is the stiffness of the automated sensor in macrophages, whose activity regulates the polarization response [23]. Toll-like receptor (TLR) signaling pathways play a significant role in the anti-infection immunity of macrophages. The TLR4 receptor and Piezo1 protein form a complex between macrophages, which are stimulated by bacterial infection or LPS, and the Ca^2+^ influx generated by the activation of Piezo1. The phosphorylation of key mammalian STE20-like protein kinases 1 (MST1) and MST2 in the calmodulin (CaM)-dependent kinases II (CaMKII) and Hippo signaling pathways is activated, which then acts on the Rac signaling pathway to regulate the release of reactive oxygen species (ROS) by mitochondria, and promote the phagocytosis and antibacterial ability of macrophages [24]. Macrophages, which reside in and infiltrate lung tissue, constantly sense external signals of mechanical stimulation, and respond through Piezo1. A mechanostimulus-immune signal pathway activated by Piezo1, in response to circulating hydrostatic pressure, activating the transcription of c-Jun N-terminal kinase (c-JUN) and upregulating endothelin-1 (EDN1), has just been reported in a journal. The endothelin receptor B (Ednrb) signaling pathway can also stabilize the expression of hypoxia irreducible Factor 1α (HIF1α) and thus promote and prolong the pro-inflammatory response process [25]. It is also suggested that Piezo1 is required for iron stress and phagocytosis in macrophages, a process that is exacerbated by overactive Piezo1, which reduces its ability to react to severe iron stress. The transport of Ca^2+^ is triggered by Piezo1, and it activates Rac1, a small GTPase that is activated by calcium, which enhances phagocytosis and regulates the cytoskeletal tissue of macrophages. It was investigated the probable mechanisms by which macrophages affect phagocyte function through the activation of Ca^2+^ and Rac1, due to excessive mechanotransduction. In conclusion, the upregulation of Piezo1 in macrophages may increase the transport of red blood cells as a direct consequence of increased hemophagocytosis [26].

One of the current mechanisms through which macrophages are activated is that, directly or through interaction with TLR receptors, Piezo1 mediates Ca^2+^ influx, controls the expression of EDN1, induces the nuclear translocation of HIF1α and NF-κB, and promotes extracellular secretion of the proinflammatory cytokines IL-6 and TNF-α. The feedback loop between Piezo1 and the polymerization of actin also has significant implications for the function of macrophages [27]. The exact mechanisms of Piezo1 and TLRs are unclear and need further research.

DCs are specialized antigen-presenting cells (APCs) that transmit stimuli of foreign antigens to T cells via different signaling pathways to guide the differentiation of different T-cell subsets. Ultimately, the outcome of the immune reaction is determined. Changes in inflammatory or mechanical stimuli, such as changes in cell-stretching stress, may activate Piezo1 signaling, which regulates the responses of innate immune cells (e.g., DCs) and triggers inflammatory responses (Figure 3). Recent studies have demonstrated that DC cells growing at a high rigidity in vitro show significantly increased proliferation, activation, and cytokine production, and a significant upregulation of glucose metabolism pathway flux, including the tricarboxylic acid (TCA) cycle and pentose phosphate pathway (PPP), compared to DC cells grown at physiological resting stiffness. Under mechanical forces, Piezo1 is proposed as a potential mechanism molecule that may be an important effector of metabolism and the function of DCs [28]. We conclude that Piezo1, the DC mechanical sensor stimulated by mechanical forces or by signals from the inflammatory response, regulates the reciprocal differentiation of Th1 and regulatory T cells (Treg cells) in inhibiting the growth of tumors. A variety of different types of stimuli exist in the tumor microenvironment, and inflammation and mechanical force may change the outcome of an immune response. One study revealed that inflammation and mechanical force can play a critical role in the differentiation of DC-directed T-cell subsets, which provides an experimental basis for a more comprehensive understanding of the tumor immune microenvironment. In addition, a mechanism for the control of cytokines secreted by Piezo1 in DCs was further investigated in this work. It is noted, however, that Piezo1 can mediate the SIRT1-HIF1α-glucose metabolism signal pathway and the calcium-ion signal-calmodulin-nuclear transcription factor NFAT signal pathway. This suggests that the ionic channel Piezo1, which is active in both inflammatory and mechano-stimulating responses, can mediate changes in ion concentration and metabolic signals in the cellular environment, altering their function and ultimately regulating the differentiation of different T-cell subsets, thus playing a key role in determining the outcome of disease. The mechano-force receptor Piezo1 regulates the differentiation of DC-mediated T-cell subsets and has promising clinical applications in antitumor immunotherapy [29].

Piezo1 significantly regulates the function of several innate immune cells, such as macrophages and DCs, but the exact mechanisms are unclear and need further investigation.

## 4. Piezo1 Affects the Function of Adaptive Immunity

**Figure 4:**
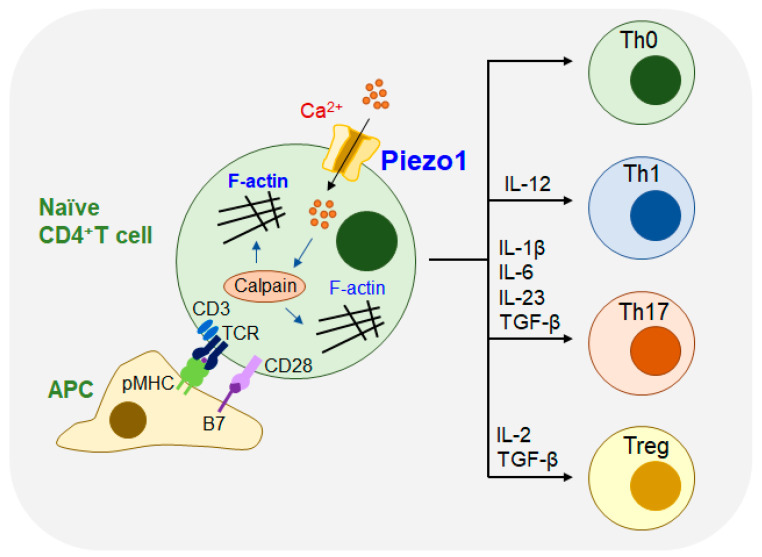
Piezo1 activates different subsets of CD4^+^ T cells. Different subsets of T cells are activated or recognized by short peptides, such as CD4^+^ T cells on antigen-presenting cells (APCs) by T cell receptor (TCR) or cross-linked antibodies immobilized on magnetic beads and induced by specific cytokines. In T cells, Piezo1 can promote extracellular Ca^2+^ influx. Piezo1-mediated Ca^2+^ influx was further induced by CD3/CD28 antibody or anti-CD3/anti-CD28 antibody. Piezo1 activates Ca^2+^ influx and calpain expression in response to mechanical stimulation, leading to cytoskeletal rearrangement. Thus, TCR recognition of peptides on major histocompatibility complex (MHC) molecules can promote traction force production and Ca^2+^ influx of T cells, ultimately leading to T cell activation.

In the adaptive immune system, mechanical forces are involved in regulating molecular interactions. Piezo1 is involved in regulating DC activation and T-cell priming [29] as shown in Figure 4.

Adaptive immune cells detect and respond to signals from mechanical stimulation from the external environment, which is crucial in regulating the process of diseases. Adaptive immune cell activation occurs primarily through the detection and discrimination of antigens by the receptors of B cells and T cells (Figure 4). In T cells, T-cell receptors (TCRs) recognize peptides expressed on the surface of APCs, which are accompanied by major histocompatibility complex (MHC) molecules. The interaction between the MHC and TCRs forms the dynamic macromolecular structure immune synapse (IS), that is located on the membrane of the cell, which results in the rearrangement of the cytoskeleton and the activation of a series of downstream signaling pathways [30].

Recent studies have shown that Piezo1 plays a major role in the activation of human T cells. In T cells, Piezo1 can facilitate extracellular Ca^2+^ influx, and cross-linking of the TCR can also accelerate Ca^2+^ influx. CD3/CD28 or anti-CD3/anti-CD28 Abs could also induce Piezo1-mediated Ca^2+^ influx. When T cells are stimulated by IS, the mechanical stimulation of Piezo1 cells activates the influx of Ca^2+^ and the unregulated expression of calpain, resulting in a reorganization of actin and cytoskeleton rearrangement. Therefore, the TCR recognizes peptides on MHC molecules, which can promote traction-force generation and Ca^2+^ influx of T cells, eventually resulting in the activation of T cells [31].

T cells react to complex mechanical signals during the immune response and differentiate into different subsets. Piezo1 is a mechanosensitive ion channel that plays an important role in the proliferation and differentiation of T cells, and is indispensable for the polarization of effector Th1 and Th17 cells. However, T cells with Piezo1 knockout have shown enhanced transforming growth factor-β (TGFβ) signaling and increased Tregs cells. Piezo1 can selectively inhibit Treg cells without affecting the function of living effector T cells, thereby reducing the incidence of autoimmune encephalomyelitis (EAE) [32].

In antitumor therapy with immune cells, CD4^+^ T cells can play a significant regulatory role in the development of effector Th1, Th2, Th17, and immunosuppressive Treg cells. Recent studies have demonstrated that the Piezo1 protein of DCs responds to inflammatory and mechanical stimulation and further regulates the differentiation of Th1 and Treg cells, to prevent tumors in the tumor microenvironment under other circumstances. The signal by Piezo1 in DCs regulates the differentiation of Th1 cells and Treg cells by governing the secretion of cytokines IL-12 and TGFβ1 and interfering with the receptors on the surface of T cells. These signaling pathways may provide novel strategies for clinical studies on the differentiation of T cells targeting DCs, which can be used in the treatment of tumor immunotherapies [29]. At present, no study has shown that Piezo1 plays a role in COVID-19 mediated lung injury, and further studies are needed.

## 5. Piezo1 Impacts Tumor Progression

In a wide variety of scenarios, the mechanical properties of the tumor are very different from those of the host tissue. Stimulation of reflex signals regulates the progression of cancer by affecting tumor cells and their microenvironment, and altering tumor cell genesis, proliferation, adhesion, migration, and invasion. Cancer cells detect mechanostimulatory signals, transmuted into the cellular response by means of the mechanosensitive ion channel Piezo1, involved in mediating the infiltration of tumor cell Ca^2+^ into the cells [33]. The hardness of solid tumor tissue is not the same as that of normal tissue. Therefore, mechanical stimulation signals affect normal physiological activity and cause a set of pathological effects through the Piezo1 ionic channel, which is sensitive to mechanical stimulation. Then, in the process of tumor migration, tumor cells shed, escape from blood vessels, and migrate to the various tissues and organs of the body, during which corresponding mechanical stimulation signals are generated, resulting in changes in the automated signals of the system. Ultimately, changes in reflex signals can induce cancer and affect the pathologic formation of tumors. Further studies have demonstrated that Piezo1 is involved in several cancer processes, including the proliferation of tumor cells and the onset of cell death in the tumor.

Piezo1 has been shown to be a potential proto-oncogene in stomach cancer, one of the most common malignancies (Table 2). The detection of Piezo1 upregulation in primary gastric cancer tumor samples suggests a poor prognosis in gastric cancer patients. When the expression of Piezo1 is slowed, obvious recompositing of the cytoskeletal system and other morphologic changes in gastric cancer cells lead to the invasion of gastric cancer cells. In the development and progression of cancer of the gastric duct, Piezo1 regulates epithelial homeostasis, promoting the proliferation, migration, and invasion of cancer cells [34]. Epithelial cells are thought to act as a biochemical and physical barrier to maintain epithelial homeostasis in the organs they envelop. Under normal physiological circumstances, the number of epithelial cells is controlled within a certain range to protect the physiological function of the tissues, and to prevent the occurrence of tumors. After the deletion of Piezo1, the mechanosensitive ion channel regulates the density of cells during the formation of tumors, either by the division of cells or the squeezing of excess cells. Piezo1 allows cancer cells without restriction to continue multiplying, regardless of whether they get away with contact inhibition. Trifolium factor family 1 (TFF1), which is a member of the tuff domain peptide family, is involved in the repair and migration of cells in the epithelium. Recent studies have confirmed that TFF1, but not TFF2 or TFF3, binds and colocalizes Piezo1 in the cytoplasm, suggesting that this interaction may be a therapeutic target for inhibiting the invasion and metastasis of gastric cancer, and that this interaction has a significant role in mediating the migration of cancer cells in the gastric cavity, which suggests that it may be an effective therapeutic target to inhibit the invasion of the gastric cavity, and the metastasis of the cancer [35].

In addition to gastric cancer, there is evidence that mechanical stresses and elevated mechanical signals promote the metastasis of malignant colon tumors. The upregulated expressions of Piezo1 and HIF-1α are closely linked to poor prognosis in patients with colon cancer. The overexpression of Piezo1 promotes the migration of colon cancer cells and downregulates the mitochondrial membrane potential. In addition, the deletion of Piezo1 inhibits the expression of HIF-1α and vascular endothelial growth factor (VEGF). However, when HIF-1α expression is deleted, Piezo1-overexpressing cells do not recover their migration function. It has also been associated with elevated expression of mitochondrial calcium unipolar (MCU) and VEGF. The results indicate that the signaling axis of Piezo1-MCU-HIF-1α-VEGF mediates the metastasis of colon cancer cells [36].

Piezo1 plays an increasingly physiological role in hepatocellular carcinoma (HCC). A market opportunity study update confirms that Piezo1 is highly expressed in HepG2 cell lines, and the absence of Piezo1 promotes the proliferation, migration, and apoptosis of liver cancer cells. The entry of Ca^2+^ into hepatocellular carcinoma cells, caused by the piezoelectric ceramic-specific activator Yoda1, activates the phosphorylation of Jun N-terminal kinases (JNK), mitogen-activated protein kinase (p38) and extracellular regulated protein kinases (ERKs) and activates the mitogen-activated protein kinase (MAPK) pathway in a time- and dose-dependent manner. The activation of Piezo1 regulates the nuclear translocation of Yes-associated protein (YAP) and the regulation of downstream genes, and MAPK signals regulate the Yoda1-induced YAP signaling pathway. Correspondingly, in Piezo1-knockout mice, the growth and development of liver cancer tumors are prevented. In summary, the potential target of Piezo1 in drug therapy could be used by organisms to suppress the growth of hepatic tumors through the Piezo1-MAPK-YAP signaling pathway [37].

Lung cancer is one of the most widespread and deadly diseases, and the current treatment regimen is very difficult to cure malignant lung cancer, with an overall survival rate of approximately ten percent. The loss of Piezo1, located in the endoplasmic reticulum (ER), inactivates the endogenous beta1 integrity affinity, suppressing cell adhesion. Scientists have found that the loss of Piezo1 reduces the hypothecate dependence of cell migration. A decrease in the expression of Piezo1 and a decrease in integrin-dependent migration have been observed in small cell lung cancer (SCLC), so it is believed that the loss of Piezo1 expression may be a cause of the increased invasion and metastasis of lung cancer cells [38]. The results of next-generation sequencing analysis of lung cancer (LC) have shown that the function of Piezo is closely related to the occurrence and development of LC. The mRNA expression of Piezo1 and Piezo2 is significantly lower in tumor tissues of non-small cell lung cancer (NSCLC) than in neighboring nontumor tissues. Studies have shown a strong correlation between higher levels of mRNA in the Piezo channel and better overall survival in patients presenting with NSCLC but not in patients presenting with lung squamous cell carcinoma (LUSC). The Piezo1 protein in small-cell lung cancer cells could significantly stimulate the migration of tumor cells in vitro and the growth of tumors in vivo. These results will allow us to further explore the pathological mechanism of NLCLC and offer more effective treatments for patients with NLCLC [39].

Breast cancer cells can migrate from primary tumors in a variety of ways from the primary tumor. One of the main ways is the migration of deformed cells, which is dependent on the formation of bubbles. The use of Yoda1, which is activated by a mechanically sensitive calcium channel piezoelectric Piezo1, also reduces the thrombinase-induced bubbles, which are reduced in cells where Piezo1 is knocked out. Piezo1 activation inhibits thrombin-induced phosphorylation of the ezrin, radixin, and moesin (ERM) proteins, which are involved in the foaming process. In the development of new drugs, Piezo1 activation could offer a novel way to inhibit thrombinase-induced blistering in breast cancer cells [40].

Oral squamous cell carcinoma (OSCC) cells are susceptible to a constantly changing external environment that affects the physiological activity of tumor cells. Previous studies have shown that the YAP signaling pathway is involved in the proliferation of OSCC cells. However, the Ca^2+^ channel protein Piezo1, a transcriptional target molecule for YAP signaling, is associated with OSCC cell growth. The upregulation of Piezo1 expression is necessary for the transport of piezoelectric receptor agonist-dependent Ca^2+^ and the proliferation of OSCC cells. The results reveal that the organism promotes OSCC cell growth through the YAP-Piezo1 signaling pathway [41].

In pancreatic ductal adenocarcinoma (PDA), the Piezo1 channel has been detected in myeloid cells as a sensor for mechanical stimulation in tumor cells. The elimination of Piezo1 in myeloid cells could prevent the development of cancer and enhance survival rates for multimicrobiological sepsis. Scientists have concluded that reflex stimulation signals promote the migration of myeloid cells, which is driven by Piezo1, by suppressing the retinal blastoma gene Rb1. Piezo1 blocks the expression of Rb1 by controlling histone decarboxylase 2. In conclusion, Piezo1 may serve as a checkpoint of the immune system that drives immunosuppression in PDAs [42].

Cancer metastasis is an important cause of cancer death. Recent studies have demonstrated new methods for the prevention of cancer metastatic [43]. Cancer cell metastasis is a process by a variety of mechanical forces. Cancer cells can be stimulated by the calcium-ion channels of Piezo1, by converting mechanical stimuli into electrochemical signals. It has been shown that the activation of Piezo1 can promote the migration of Stomach cancer, Colon tumors, HCC, breast cancer cells, and PDA [34,36,37,40,42]. However, the results of studies in NSCLC showed that the down-regulation of Piezo1 expression promoted tumor migration [39]. The different effects of Piezo1 in promoting metastasis in different types of cancers indicate the complex diversity in the progression of the cancer. This suggests that inhibition or activation of Piezo1 could be a potential target for the treatment of cancer. But different organs exist in different environments and respond to different signals and mechanical forces. In some cases, the downregulation of Piezo1 may be a target to prevent cancer cell metastasis, but in other cases, the promotion Piezo1 expression effectively prevented cancer cell metastasis, which suggests that the specific roles of mechanical force signaling and Piezo1 in cancer metastasis need to be further investigated.

## 6. Concluding Remarks

As a mechanosensitive ion channel protein, Piezo1 plays a key role in the inflammatory response caused by mechanical stress imbalance. Under continuous and repeated mechanical force stimulation, Piezo1 can convert mechanical signals into an intracellular inflammatory response, which leads to chronic tissue inflammation. Current studies have shown that Piezo1 drugs can be used as potential targets for the treatment of mechanical stress-related chronic inflammatory diseases. Both mechanical force signals and inflammatory responses are dynamic processes that change with time. Piezo1 can inhibit the occurrence of inflammatory events at the early stage of mechanical injury, which is a powerful means to prevent chronic inflammation [44].

Transient receptor potential vanilloid 1 (TRPV1) is an ion channel expressed on sensory neurons, which has similarities with Piezo1 and can trigger cation (Ca^2+^, Na^+^) influx. TRPV1 acts as a receptor for noxious stimuli such as heat and pain, which can help the body avoid pain. TRPV1 activation has also been linked to chronic inflammation. TRPV1 is also expressed in non-neuronal areas such as the lung and bladder, affecting the disease progression of cystitis and asthma [45].

Cancer is a major disease that threatens human health. Cellular immunotherapy brings new hope to immunotherapy against cancer. Piezo1 plays a crucial role in the regulation of tumor growth and metastasis, but it has different effects on the function and differentiation of innate and adaptive immune cells, which is different from the supervisory mechanism of Piezo1 on tumor cells themselves. The tumor microenvironment is of great significance for the growth of the tumor. The natural stimuli in the microenvironment of tumors are diverse, including inflammatory stimuli and mechanical stimuli. The tumor-immunomodulatory effects of Piezo1 on macrophages, DCs, and T cells are an area worthy of further investigation for the treatment of immune cell antitumor therapy. The study of the Piezo1 target of specific tumors in the microenvironment may also reveal that different immune cells also play different roles in the regulation of the immune system, and it is necessary to further confirm the potential benefits of different anti-Piezo1 immunotherapies. Therefore, further study on the regulation and mechanism of Piezo1 in different immune systems and in the microenvironment of tumors will provide an essential basis for the study of immunotherapy in tumors.

## Figures and Tables

**Table 1:** Classification of ion channel families.

Mechanically Gated Ion Channels	Ligand-Gated Ion Channels	Voltage-Gated Ion Channels
Msc (MscL, MscS, Msc-like)	nAChRs	VGSCs (Na^+^)
K2P (Trek1, Trek2, Traak)	GlyRs	VGCCs (Ca^2+^)
Piezo (Piezo1, Piezo2)		VGPCs (K^+^)
OSCA		
TMEM63A/B		
TRP (NOMPC, TRPV4)		
MET		
DEG (Mec4/Mec10, ENaCs, ASICs)		

Mechanosensitive channel conductance (Msc); mechanosensitive channel small conductance (MscS); mechanosensitive channel large conductance (MscL); two-pore potassium channel (K2P); hyperosmolality-gated calcium-permeable channels (OSCA); transmembrane protein 63 (TMEM63); transient receptor potential (TRP) channels; no mechanoreceptor potential C (NOMPC); transient receptor potential vanilloid-4 (TRPV4); mechano-electrical transduction (MET) channel; epithelial Na^+^ channels (ENaCs); degenerin (DEG); acid-sensing ion channels (ASICs); nicotinic acetylcholine receptors (nAChRs); glycyl-tRNA synthase (GlyRS); voltage gated sodium channel (VGSC); voltage-gated calcium channels (VGCCs); and voltage-gated potassium channels (VGPCs).

**Table 2:** Functional modulation of Piezo1 in cancer.

Disease	Target	Mechanism	Effect on Disease/Clinical
Stomach cancer	TFF1	TFF1, which binds and colocalizes Piezo1 in the cytosol, mediates migration of cancer cells in the gastric cavity	Piezo1 regulates the proliferation, migration, and invasion of stomach tumors [34]
Colon tumors	HIF-1α	Piezo1-MCU-HIF-1α-VEGF signaling axis mediates the metastasis of colon cancer cells	Overexpression of Piezo1 promotes the migration of colon cancer cells [36]
Hepatocellular carcinoma	JNK p38 ERK	The Piezo1-MAPK-YAP signaling pathway may inhibit the growth of hepatocellular tumors	Absence of Piezo1 promotes the proliferation, migration, and apoptosis of liver cancer cells [37]
Lung cancer	Piezo2	The mRNA expression of Piezo1 and Piezo2 is significantly lower in tumor tissues of non-small cell lung cancer (NSCLC)	Piezo1 in NLCLC could significantly stimulate the migration of tumor cells in vitro and the growth of tumors in vivo [38,39]
Breast carcinoma	Ezrin, radixin and moesin (ERM)	Piezo1 activation inhibits thrombin induced phosphorylation of ERM	Piezo1 is an inhibitor of thrombinase-induced blistering in breast cancer cells [40]
Oral squamous cell carcinoma (OSCC)	Yes-associated protein YAP	The organism promotes OSCC cell growth through the YAP-Piezo1 signaling pathway	Upregulation of Piezo1 expression is necessary for OSCC cell proliferation [41]
Pancreatic ductal adenocarcinoma	Rb1	Piezo1 inhibits Rb1 expression by regulating histone decarboxylase 2	Elimination of Piezo1 in myeloid cells could prevent cancer [42]

Functional relevance of Piezo1 in cancer. In recent years, a number of studies have shown that Piezo1 plays an important role in regulating the occurrence and development of various tumors, such as stomach cancer [34], colon tumors [36], hepatocellular carcinoma [37], lung cancer [38,39], breast carcinoma [40], oral squamous cell carcinoma [41], and pancreatic ductal adenocarcinoma [42]. Table 2 shows various signaling pathways related to the induction of key regulators of Piezo1 channel proteins, in response to mechanical stimulation, which provides new research ideas for subsequent tumor treatment.
